# Immunohistochemical expression of melan-A and tyrosinase in uveal melanoma

**DOI:** 10.1186/1477-3163-6-6

**Published:** 2007-04-20

**Authors:** Bruno F Fernandes, Alexandre N Odashiro, Vinicius S Saraiva, Patrick Logan, Emilia Antecka, Miguel N Burnier

**Affiliations:** 1 Department of Ophthalmology and Pathology, The McGill University Health Center & Henry C, Witelson Ocular Pathology Laboratory, Montreal, Canada; 2 Department of Ophthalmology, Federal University of São Paulo, UNIFESP/EPM, São Paulo, Brazil

## Abstract

**Background:**

Melan-A and tyrosinase are new immunohistochemical markers that can be used in the diagnosis of melanocytic lesions. The aim of this study was to investigate the correlation between radiotherapy or clinicohistopathological parameters and the expression of melan-A and tyrosinase in uveal melanoma.

**Methods:**

Thirty-six enucleated cases of uveal melanoma were studied. The formalin-fixed, paraffin-embedded specimens were immunostained with monoclonal antibodies against melan-A and tyrosinase. The samples were classified as either positive or negative. The chi-square or the Student-t tests were used to test for the correlation of the expression rates of melan-A and tyrosinase with clinico-pathological parameters.

**Results:**

Melan-A and tyrosinase were positive in 33 (91.7%) and 35 (97.2%) of the specimens, respectively. There was no significant association between the expression of melan-A or tyrosinase and radiotherapy or any clinico-pathological parameter. All specimens were positive for at least one of the immunohistochemical markers.

**Conclusion:**

To the best of our knowledge this is the first study concluding that the expression of melanocytic markers such as melan-A and tyrosinase is not influenced by radiotherapy or any clinico-pathological parameter. Moreover, when tyrosinase and melan-A are used together, 100% of the formalin-fixed, paraffin-embedded uveal melanoma samples tested positive for one of those markers.

## Background

Uveal melanoma is the most common primary intraocular malignancy in adults, with an incidence of 5–7 new cases per million people per year.[1] Over the past few decades, treatment of the primary tumor has drastically improved and radiotherapy has replaced enucleation as the preferred treatment of the primary tumor.[2] However, despite the growing success of treating the eye, the systemic prognosis has not improved: the 5-year survival rates have remained practically unchanged in recent decades, ranging from 77 to 84% from 1973 to 1993, without a statistically significant variation[2,3]. Tumor-related death is mainly due to liver metastasis, which is usually detected several years after the diagnosis and treatment of the primary tumor[4].

The melan-A protein is a melanocytic differentiation antigen, product of the MART-1 gene, and is thought to be specific for melanocytic cells.[5] It was found to be a useful addition to antibody panels for cutaneous melanocytic lesions.[6] Tyrosinase is an enzyme involved in the initial stages of melanin biosynthesis in melanocytes and melanoma cells and, for that reason, is also considered a biochemical marker of melanocytes.[7] A two-marker polymerase chain reaction (PCR) using melan-A and tyrosinase has been described for the detection of Circulating Malignant Cells (CMCs) in the peripheral blood of patients with skin melanoma.[8] The combination of these two markers was also described for the detection of CMCs in uveal melanoma.[9,10] However, only a few studies evaluated the co-expression of these immunohistochemical markers in primary uveal melanomas.[11-14] To the best of our knowledge, a study investigating the influence of radiotherapy on the expression of markers of melanocytic differentiation has never been done in uveal melanoma.

The aim of this study was to investigate the expression of melan-A and tyrosinase in uveal melanoma, and the correlation with radiation therapy or clinicopathological parameters.

## Methods

### Patients

Thirty-six patients with uveal melanoma were included in the study based on the availability of representative tissue and clinicopathological data. Subjects' pathological reports and Cancer Registry entries were reviewed to provide the following information: age at diagnosis, gender, previous ocular radiation therapy, largest tumor dimension (LTD), cell type, lymphocytic infiltration and presence of closed vascular loops.

The cell type was classified according to the modified Callender's classification of uveal melanoma [15]. Tumors composed of only spindle cells were classified as spindle, whereas tumors containing spindle and epithelioid cells were classified as mixed. The LTD, in millimeters, was measured by ultrasound prior to treatment. The classification of lymphocytic infiltration and closed vascular loops was done as described elsewhere. [16]

### Tissue samples

Thirty-six enucleated eyes containing tumor tissue were routinely fixed in 10% buffered formalin and subsequently paraffin-embedded. Paraffin blocks were retrieved from the Henry C. Witelson Ocular Pathology Laboratory and Registry, McGill University, Montreal, Quebec, Canada.

### Immunohistochemistry

Immunostaining was performed according to the avidin-biotin complex technique. Briefly, 4 μm thick sections, were deparaffinized in xylene and rehydrated through graded ethanol washes. Endogenous peroxidase activity was blocked with a 10-min wash with 3% hydrogen peroxide in methanol. Heat antigen retrieval was performed with microwave treatment in citrate buffer (pH 6.0). Non-specific binding was blocked with a 30-min wash with 1% bovine serum albumin (BSA) in Tris-buffered saline (TBS, pH 7.6).

Sections were incubated overnight with immunohistochemistry-specific rabbit antibody for melan-A (NCL-L-Melan-A, diluted 1: 25, Novocastra Laboratories Ltda, United Kingdom) and tyrosinase (NCL-TYROS, diluted 1:25, Novocastra Laboratories Ltda, United Kingdom).

Following incubation with primary antibody at 4°C, sections were incubated with biotinylated goat anti-rabbit secondary antibody (diluted 1:500; DAKO, Mississauga, Ontario, Canada) for 30 min at room temperature. Sections were then incubated with horseradish peroxidase-conjugated streptavidin-biotin complex (DAKO) for 30 min at room temperature. Immunostaining was visualized using the 3-amino-9-ethylcarbazole (AEC) chromogen (DAKO). Sections were counterstained with hematoxylin and cover-slipped.

The omission of primary antibody and the use of non-immune serum (0.1% BSA in TBS) served as a negative control. Skin melanoma served as the positive control for both antibodies.

### Microscopic classification

Two independent ophthalmic pathologists analyzed the slides by light microscopy and the final interpretation was based on agreeing assessments. Samples were classified into two categories: negative (if less than 10% of the tumor cells displayed immunostaining) and positive (if more than 10% of tumor cells displayed distinct immunostaining, irrespective of the staining intensity). [12]

### Statistical analysis

The chi-square test was used to test the correlation of expression with gender, radiotherapy, cell type, lymphocytic infiltration and presence of vascular loops while the student-T test was used for age and largest tumor dimension. A *P*-value of less than 0.05 was considered to be statistically significant.

Data accumulation was acquired in accordance with Country and Provincial laws, and the tenets of the Declaration of Helsinki.

## Results

### Patients

The sample studied was composed of 36 patients, 23 males (63.9%) and 13 females (36.1%). Age at diagnosis was 65 ± 11 years (mean ± standard deviation). Twenty-one patients were treated with primary enucleation while 15 were treated with radiotherapy and enucleation. The time period between radiotherapy and enucleation was 52.4 ± 44.1 months (mean ± standard deviation). Regarding cell type, 3 were classified as spindle and 33 as mixed cell type uveal melanomas. LTD was 15 ± 4.9 mm (mean ± standard deviation). Lymphocytic infiltration and closed vascular loops were present in 8 (22.2%) and 10 (27.8%) patients, respectively.

### Expression of melan-A and tyrosinase

**Figure 1:**
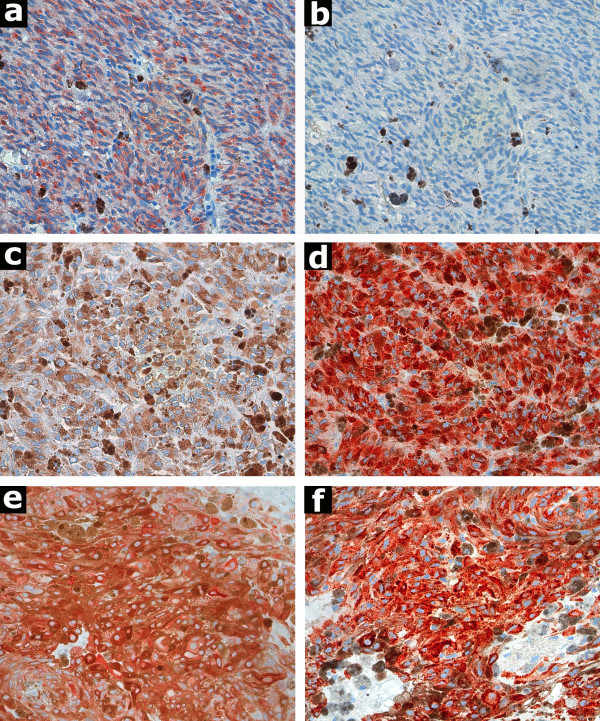
Photomicrographies. Choroidal melanoma showing positive immunostaining for melan-A (A) and negative for tyrosinase (B) Another patient now showing negative immunostaining for melan-A (C) and positive for tyrosinase (D). Choroidal melanoma enucleated after failure of treatment with brachytherapy. Positive Immunostaining for melan-A (E) and tyrosinase (F). (red chromogen, ×400).

Immunoexpression of melan-A and tyrosinase was cytoplasmatic (Fig. 1). The staining pattern was diffuse in the positive samples for both markers. Melan-A and tyrosinase were positive in 33 (91.7%) and 35 (97.2%) of the specimens, respectively.

All specimens were positive for at least one of the immunohistochemical markers.

### Correlation with clinicopathological parameters

There was no observable association between the expression of each melanocytic marker and gender, radiotherapy, cell type, lymphocytic infiltration and presence of closed vascular loops (p > 0.05). Age and LTD were not correlated with the expression of melan-A either. The statistical analysis for age and LTD could not be performed for tyrosinase given the small number of negative results (Tables 1 and 2).

**Table 1:** Correlation of the expression of melan-A with clinico-pathological parameters

	**Positive (33)**	**Negative (3)**	**Test**	**p-value**
**Age at diagnosis (years, mean ± standard deviation)**	65 ± 10.8	60 ± 14.4	Student-t	0.43
**Sex (n)**				
**Male**	21	2	chi-square	0.92
**Female**	12	1		
**Radiotherapy (n)**				
**yes**	14	1	chi-square	0.76
**no**	19	2		
**Cell type (n)**				
**Spindle**	3	0	chi-square	0.59
**Mixed**	30	3		
**Largest tumor dimesion (mm, mean ± standard deviation)**	14.8 ± 5.1	12 ± 2.8	Student-t	0.46
**Lymphocytic infiltration (n)**				
**present**	8	0	chi-square	0.33
**absent**	25	3		
**Closed vascular loops (n)**				
**present**	10	0	chi-square	0.26
**absent**	23	3		

**Table 2:** Correlation of the expression of tyrosinase with clinico-pathological parameters

	**Positive (35)**	**Negative (1)**	**Test**	**p-value**
**Age at diagnosis (years, mean ± standard deviation)**	65 ± 11.2	67	n/a	n/a
**Sex (n)**				
**Male**	22	1	chi-square	0.45
**Female**	13	0		
**Radiotherapy (n)**				
**yes**	15	0	chi-square	0.39
**no**	20	1		
**Cell type (n)**				
**Spindle**	3	32	chi-square	0.76
**Mixed**	0	1		
**Largest tumor dimesion (mm, mean ± standard deviation)**	15 ± 4.9	8.5	n/a	n/a
**lymphocytic infiltration (n)**				
**present**	8	27	chi-square	0.59
**absent**	0	1		
**Closed vascular loops (n)**				
**present**	10	25	chi-square	0.53
**absent**	0			

### Non-neoplastic ocular tissues

Melan-A stained normal uveal melanocytes in a variable fashion. A similar pattern was noted with tyrosinase staining, albeit with less intensity. No immunostaining of any other ocular structure was seen with either marker.

## Discussion

HMB-45 and S-100 are the two most common markers used for the diagnosis of uveal melanoma.[17] HMB-45 recognizes the protein gp100[18] and is more specific than S-100, which is positive in other non-melanocytic tumors[19]. Melan-A and tyrosinase were recently described as markers of melanocytic differentiation. Studies with formalin-fixed, paraffin-embedded sections of cutaneous melanoma showed positive results for melan-A and tyrosinase in 97% and 90% of the cases, respectively [20].

De Vries et al[14] studied the expression of melan-A and tyrosinase in cryostat sections of 32 cases of uveal melanoma and all of them were positive. Four specimens of uveal metastatic lesions also stained positive for both markers. However, it was uncertain whether the same positive results could be achieved in formalin-fixed, paraffin-embedded specimens. In addition, clinical data of the patients, including method of treatment, was not available.

Posteriorly, formalin-fixed, paraffin-embedded sections of uveal melanoma were studied for the expression of melan-A and tyrosinase [11-13]. High rates of positive results were seen for both markers and the staining was not influenced by cell type. However, as in the previous study, no clinical information regarding any treatment prior to enucleation was provided.

Ionizing radiation is known to induce a myriad of pathological changes in different tissues [21]. At a molecular level, exposure of cells to ionizing radiation results in immediate and widespread oxidative damage. Following these immediate biochemical events, a wide range of covalent damage is induced in cellular DNA, including strand breaks, base and sugar damage, cross-links between DNA strands and DNA-protein bonds. This DNA damage leads to cell cycle arrest and/or apoptosis [22]. Although *in vitro *studies have shown that radiation causes chromatid and isochromatid chromosome breaks in melanoma cells. [23], uveal melanoma is notoriously highly radioresistant [24].

An analysis of melanoma cell type, before and after radiotherapy, was performed by Char et al [25] in patients that had a preradiation fine needle aspiration biopsy (FNAB) and later required an enucleation. In a group of 35 uveal melanoma patients, 20 had no sequential change in the cell type, 14 had increased malignancy based on sequential studies, and 1 specimen showed a change from a mixed to a predominantly spindle B cell type. The expression of melanocytic markers was not investigated.

To the best of our knowledge this is the largest sample of formallin-fixed, paraffin-embedded specimens of uveal melanoma, studied for the co-expression of melan-A and tyrosinase. Moreover, this is the first time that the expression of those markers was correlated with clinico-histopathological parameters other than cell type. We showed that all samples were positive for at least one of the markers, regardless of any tumor characteristic or previous treatment with radiation.

The conclusions of our study bring two important implications. First, the association of these two markers is useful to confirm the diagnosis in atypical cases of uveal melamoma, because in every sample at least one of them will be positive. The second implication is related to the detection of CMCs using RT-PCR for melan-A and tyrosinase. It is important to note that the primary tumor consistently expresses those markers independent of any tumor feature and even in patients that were treated conservatively with radiation. Consequently, our results suggest that the use if both aformentioned markers together will ensure that CMCs, if present, will not avoid detection.

Given the overall high expression of both markers and the small number of negative results, a statistical analysis concerning the timing of radiotherapy before enucleation could not be performed. However, our sample was composed of patients that received radiotherapy as soon as one month prior to the surgery and others that were enucleated almost ten years after. Although a higher number of uveal melanoma specimens should be studied first, we believe that it is unlikely that the time of radiotherapy plays any role in the expression of the melanocytic markers herein studied.

In agreement with previous studies, our results support the evidence that an immunohistochemical panel containing these two markers is highly effective in the diagnosis of uveal melanoma.

## Competing interests

The author(s) declare that they have no competing interests.

## Authors' contributions

BFF was the primary investigator and responsible for the writing of the manuscript. ANO did the histopathological evaluation and drafting of the manunscript. VS did the statistical analysis and revision of the manuscript. PL participated in the design of the study and performed the critical revision of the manuscript. EA did was the immunohistochemistry and helped to draft the manuscript. MNBJr. participated in its design and coordination and helped to revise the manuscript. All authors read and approved the final manuscript.
